# Tongluo Yishen Decoction Ameliorates Renal Fibrosis *via* Regulating Mitochondrial Dysfunction Induced by Oxidative Stress in Unilateral Ureteral Obstruction Rats

**DOI:** 10.3389/fphar.2021.762756

**Published:** 2021-10-12

**Authors:** Qi Jia, Lin Han, Xiaoyu Zhang, Wenning Yang, Yushan Gao, Yifan Shen, Bing Li, Shuyan Wang, Mingzhen Qin, Scott Lowe, Jianguo Qin, Gaimei Hao

**Affiliations:** ^1^ Department of Nephropathy, Dongfang Hospital, Beijing University of Chinese Medicine, Beijing, China; ^2^ School of Basic Medicine, Beijing University of Chinese Medicine, Beijing, China; ^3^ School of Chinese Materia Medica, Beijing University of Chinese Medicine, Beijing, China; ^4^ Emergency Department, Shanghai Municipal Hospital of Traditional Chinese Medicine, Shanghai University of Traditional Chinese Medicine, Shang Hai, China; ^5^ Beijing First Hospital of Integrated Chinese and Western Medicine, Beijing, China; ^6^ Dongzhimen Hospital, Beijing University of Chinese Medicine, Beijing, China; ^7^ Kansas City University of Medicine and Biosciences, College of Osteopathic Medicine, Kansas City, MO, United States; ^8^ Institute of Basic Theory for Chinese Medicine, China Academy of Chinese Medical Sciences, Beijing, China

**Keywords:** tongluo yishen decoction, obstruction-induced renal fibrosis, mitochondria, oxidative stress, mitophagy, Chinese medicine

## Abstract

Tongluo Yishen (TLYS) decoction is an herb that is extensively applied for the treatment of chronic kidney disease (CKD) in traditional Chinese medicine. In this study, 37 different dominant chemical constituents of TLYS were identified. Rats with unilateral ureteral obstruction (UUO) were used as animal models, and TLYS decoction was administered orally for 14 days. TLYS decoction reduced the levels of renal function indicators, serum creatinine levels and blood urea nitrogen levels and alleviated renal pathological changes. Gene Ontology (GO) and KEGG pathway analyses of RNA sequencing data showed that TLYS decoction had significant effects on biological processes, cellular components and molecular functions in UUO rats and that the phagosome (a membrane source in the early stages of autophagy), lysosome (an important component of autolysosome), and oxidation pathways (which contribute to mitochondrial function) might be related to the antifibrotic effects of TLYS decoction. Moreover, we found significant mitochondrial function impairment, including a decreased mitochondrial membrane potential (MMP) and an imbalance in mitochondrial dynamics, excessive oxidative stress, and activation of Pink1/Parkin-mediated mitophagy in UUO rats. Treatment with TLYS decoction significantly increased the MMP, normalized mitochondrial dynamics and ameliorated renal injury. Moreover, TLYS alleviated the mitophagy clearance deficiency. In conclusion, our study showed that TLYS decoction can ameliorate mitochondrial dynamics by reducing oxidative stress and regulating mitophagy, thereby relieving renal injury, protecting renal function, and reducing renal fibrosis. This study provides support for the application of and further research on TLYS decoction.

## Introduction

Chronic kidney disease (CKD) is a leading global public health issue associated with substantial comorbidities and reduced life expectancy (Mills et al., 2015). Currently, this disease affects approximately 10% of the world’s adult population, but effective treatments for its prevention and progression are lacking (Levin et al., 2017). Most patients with renal failure who progress to end-stage renal disease (ESRD) in low- and middle-income countries have little access to dialysis and kidney transplantation (Jha et al., 2013; Liyanage et al., 2015). The pathologic changes of renal fibrosis include renal interstitial fibrosis (RIF) and glomerular sclerosis, which involve epithelial injury, the inflammatory response and multiple signal transduction pathways. Studies have shown that compared with glomerulosclerosis, RIF is considered to be a crucial determinant leading to ESRD (Zeisberg and Neilson 2010). The current treatments for RIF include immunosuppressants, angiotensin-converting enzyme inhibitors, angiotensin II receptor antagonists, vitamin D and erythropoietin. Nevertheless, these treatments are still limited, and alternative therapeutic strategies are urgently needed.

Extensive studies have shown that oxidative stress is an important pathogenic mechanism of RIF, and the main cause of oxidative stress is the production of excessive reactive oxygen species (ROS) by the mitochondria (Sharma 2014; Nakanishi et al., 2019; Kitada et al., 2020). As the most important energy storage and supply sites, mitochondria are indispensable organelles in cells, but defective and aging mitochondria produce toxic ROS (Tang et al., 2021). Furthermore, ROS accumulation induces changes in mitochondrial membrane permeability and leads to the loss of mitochondrial membrane potential (MMP). Thus, clearance of damaged mitochondria is critical for cell survival to reduce the concentration of ROS. Mitophagy, a highly selective type of autophagy that eliminates damaged and aging mitochondria, is considered an important way to maintain mitochondrial quality and the stability of the intracellular environment (Li et al., 2020). In recent years, mitophagy via the PTEN-induced putative kinase 1 (Pink1)/Parkin pathways has been emphasized (Zhuet al., 2013). In normal mitochondria, Pink1 is continuously transferred to the mitochondrial intima, where it is cleaved by proteasomal degradation. However, the pathway by which Pink1 enters the mitochondrial inner membrane is blocked after loss of MMP, and Pink1 aggregates in the outer membrane of mitochondria, where it recruits and phosphorylates Parkin. Then, mitochondria are targeted for selective removal (Koyano et al., 2014; Li et al., 2020). Therefore, the clearance of damaged mitochondria by mitophagy has therapeutic potential for the treatment of RIF.

As a supplementary treatment, Chinese herbal medicine based on traditional Chinese medicine (TCM) has been widely used clinically in China for up to 2000 years. According to TCM theories, blood stasis is considered to be a key pathological factor in the pathogenesis of RIF (Guo, Li, and Rao 2019). Tongluo Yishen (TLYS) decoction has been clinically used for CKD treatment for decades and can reduce blood stasis and promote blood circulation, according to the theory of TCM. TLYS is composed of *Salvia miltiorrhiza bunge* (Danshen), *Carthamus tinctorius L.* (Honghua), *Achyranthes bidentata Bl.* (Niuxi), and *Spatholobus suberectus Dunn* (Jixueteng). These herbs or extracts are considered potential candidates for treating kidney diseases. Their bioactive properties include antioxidation (Cai et al., 2018; Do et al., 2018; Wang et al., 2020), inhibition of mitophagy (Liu et al., 2020), inhibition of epithelial to mesenchymal transition (EMT) (Li et al., 2017) and antifibrotic effects (Wang et al., 2010). However, the mechanisms of TLYS in the treatment of RIF need further study.

Hence, in this study, we focused on the effects of TLYS on renal function and mitochondrial quality. We found that TLYS ameliorated mitochondrial dysfunction, reduced oxidative stress and regulated Pink1/Parkin-mediated mitophagy in rats with unilateral ureteral obstruction (UUO). Our findings provide better insight into the molecular mechanism of TLYS as a treatment for RIF.

## Materials and Methods

### Preparation of Tongluoyishen Decoction

A total of 60 g of raw herbal pieces, including *Salvia sinica* Migo (25 g), *Achyranthes bidentata* Blume (10 g), *Salvia coccinea* Linn (15 g) and *Caulis Spatholobi* (10 g), was used. The herbal ingredients are shown in Table 1. Each herb was purchased from Dongfang Hospital affiliated with Beijing University of Traditional Chinese Medicine. The above herbs were boiled in a 10-fold volume of water at 100°C for 1.0 h. After filtration, the first extraction was boiled in an 8-fold volume of water for 0.5 h. Finally, both filtrates were mixed and concentrated to a volume of 60 ml containing 1 g/ml raw herbs. Valsartan capsules (Beijing Novartis Pharmaceutical Co., Ltd., batch number X2,375) were provided by Novartis (Bale, Switzerland).

**TABLE 1:** The herbal composition and proportion of TLYS decoction.

Scientific name	Pinyin name	Plant part used	Batch number	Herb dose (g)	Composition (%)
*Salvia miltiorrhiza bunge*	Danshen	Root	20200509	25	41.67
*Carthamus tinctorius L.*	Honghua	Flower	20111610	10	16.67
*Achyranthes bidentata Bl.*	Niuxi	Root	xf8311	15	25
*Spatholobus suberectus Dunn*	Jixueteng	Stem	20042303	10	16.67

### UHPLC-MS Analysis

TLYS decoction extract was combined with methanol and double distilled water (1:1, v/v) at 1:20, sonicated for 30 min and filtered through a 0.22 microns filtration membrane. Quality control of TLYS was performed using a UHPLC System (Dionex Ultimate 3,000, Thermo Corporation, United States) coupled with a mass spectrometer (LTQ-Oribitrap XL, Thermo Scientific). The chromatographic column was an Acquity UPLC C18 column (2.1 mm × 100 mm, 1.7 µm). The chromatographic conditions were as follows: 0.1% formic acid water (A) and methanol (B) were used as the mobile phase. The gradient elution conditions were as follows: 0–3 min, 5%–5% B; 3–45 min, 5%–75% B; 45–45.1 min, 75%–5% B; 45.1–50 min, 5% B. The column temperature was 30°C. The flow rate was 0.3 ml· min-1^,^ and the injection volume was 2 μL. A mass spectrometer equipped with an electrospray ionization source was used for both positive and negative ion mode with the mass range of 120–1800 m*/z*. The ionization voltages were 3500 V (positive mode) and 3000 V (negative mode), the capillary temperature was 320°C, the sheath gas flow rate was 35 arb and the auxiliary gas flow rate was 10 arb. XCMS software was used to import mass spectra. Peak integration, peak extraction, peak alignment, peak identification and retention time correction were carried out. Material identification of peaks was conducted with information collected from the databases and literature.

### Animal Model and Experimental Design

Male Sprague-Dawley (SD) rats (*n* = 15 per group, 180–200 g, 7–8 weeks of age) were provided by Beijing Vital River Laboratory Animal Technology Co., Ltd. (certificate number: SCXK (Beijing) 2016–0,006). The animal operation in this study was carried out according to the “Guiding Principles in the Use and Care of Animals” published by the US National Institutes of Health (NIH Publishing, No. 85–23, revised in 1996). This procedure was completed under the supervision of the Laboratory Animal Ethics Committee of Dongfang Hospital affiliated with Beijing University of Chinese Medicine (permit no. 202004). All animals were kept in a clean room at 22 ± 2°C and had free access to water and food.

As previously described, the UUO model was established in SD rats by ligation of the left ureter and sacrifice 14 days later (Masaki et al., 2003). In short, the rats were anesthetized by intraperitoneal injection of pentobarbital (50 mg/kg body weight); the left ureter was exposed via a midline incision and was ligated at two points with 4–0 silk sutures. The sham group underwent identical surgical procedures except for ligation of the ureter. The rats were randomly divided into four groups as follows: sham group, UUO group, TLYS group, and valsartan group. In the sham group and the UUO group, an equal volume of physiological saline was administered. The adult daily dosage for TLYS was 60 g. The daily dosage of TLYS in rats was calculated to be 7.8 g/kg by a correction factor equal to the human-rat body surface area ratio (6.3) (Xuan et al., 2021). The valsartan group was given 30 mg/kg/d of valsartan intragastrically.

### Measurement of Serum Creatinine (Scr) and Blood Urea Nitrogen (BUN)

The Scr and BUN levels were measured with a creatinine assay kit (C011-1, Nanjing Jiancheng Bioengineering Institute, Nanjing, China) and BUN assay kit according to the manufacturer’s instructions (C013-1, Nanjing Jiancheng Bioengineering Institute, Nanjing, China).

### Histological Examination

Six kidneys from each group were immediately fixed with 10% formalin, dehydrated, embedded in paraffin, and sectioned to a thickness of 5 µm. These sections were then stained with hematoxylin and eosin (H&E) and Masson’s trichrome. The kidney injury score was determined based on tubular atrophy and degeneration, renal papillary necrosis, interstitial inflammation, and fibrous hyperplasia as previously reported (Debelle et al., 2002).

### Immunohistochemistry (IHC) Staining

Five-micron thick paraffin-embedded kidney sections were deparaffinized, followed by antigen retrieval in ethylenediaminetetraacetic acid (1 mM). The samples were blocked with 0.3% H_2_O_2_ in methanol and 5% BSA. Kidney sections were incubated with α-SMA (1:200, 14395-1-AP, Proteintech, United States) and TGF-β1 (1:200, ab92486, Abcam, United States) primary antibodies overnight at 4°C, followed by horseradish peroxidase (HRP)-conjugated secondary antibodies (PV9001, Beijing Zhongshan Jinqiao Biotechnology Co., Ltd., Beijing). The reaction was visualized with DAB staining using a Leica Aperio Versa 8 system (Leica, Wetzlar, Germany). The cumulative optical density of the area of interest analysis was calculated using ImageJ software.

### RNA Sequencing (RNA-Seq) Analysis

Total RNA of rat kidney cortical tissue (three samples per group) was extracted using TRIzol (Invitrogen, Carlsbad, CA, United States). After total RNA extraction, eukaryotic mRNA was enriched with oligo (dT) beads, and rRNA-enriched prokaryotic mRNA was removed with a Ribo-Zerotm magnetic kit (Epicentre). Then, fragmentation buffer was used to fragment the enriched mRNA fragments into short fragments, which were reverse-transcribed into cDNA with random primers. The second strand of cDNA was synthesized by DNA polymerase I, RNase H, dNTP and buffer. The cDNA fragment was then purified with a QIAquick PCR extraction kit, the ends were repaired, poly (A) was added and the fragments were attached to the Illumina sequencing adapter. The ligation products were detected by agarose gel electrophoresis, PCR amplification and Illumina HiSeqTM 2,500 sequencing.

First, the raw data were filtered, and the clean data obtained after filtering were compared to the reference genome of the species. Second, the expression level of each gene was calculated according to the comparison results. On this basis, differential expression analysis, enrichment analysis and clustering analysis of the samples were further performed. Finally, we used DESeq for gene expression analysis and screening of differentially expressed genes as follows: multiple expression differences | log2FoldChange |>1, significance *p*-value<0.05. The Pearson correlation coefficient between all samples was calculated using the function cor, and hierarchical clustering was performed using the hclust function in the stats package in R software. Then, GO function and KEGG pathway enrichment analyses were performed on the differentially expressed genes (DEGs).

### Determination of the Mitochondrial Membrane Potential

Mitochondria were extracted from renal tissue using a mitochondrial extraction kit (C3606, Beyotime, China). Briefly, fresh kidneys were harvested, cut into small pieces, and washed thrice with precooled PBS. After digestion with trypsin, the renal tissues were homogenized in mitochondrial isolation reagent using a Dounce tissue grinder on ice. The homogenate was then centrifuged at 600 *g* for 10 min, and the supernatant was centrifuged at 1,500 *g* for 15 min to isolate the mitochondria. The change in MMP was measured by the JC-1 fluorescent probe, and the JC-1 red/green fluorescence intensity ratio was used to represent MMP. Fresh isolated mitochondria were incubated with 10 μg/ml JC-1 (at 37°C for 20 min), and the fluorescence intensity was measured by a Synergy H1 fluorometer microplate reader (BioTek, United States).

### Renal Biochemical Marker Analysis

Renal tissues were homogenized followed by ultrasonic disruption to obtain renal tissue homogenates and then centrifuged at 3,000 rpm for 15 min at 4°C. Dihydroethidium (DHE) fluorescence was used to detect the level of ROS in rat renal tissues. Detection of the malondialdehyde (MDA) level as well as the superoxide dismutase (SOD) and glutathione peroxidase (GSH-Px) activities was performed according to the instructions provided by Shanghai Biyuntian Biotechnology Co., Ltd. (Shanghai, China).

### Immunofluorescence Staining

Frozen sections were used to assess colocalization of Pink1 and TOM20. Kidney sections were blocked with 5% bovine serum albumin for 30 min at room temperature, followed by incubation overnight at 4°C with primary antibodies against Pink1 (1:200, SC517353, Santa Cruz, United States) and TOM20 (1:200, 11802-1-AP, Proteintech, United States). After the samples were washed with PBS, an Alexa Fluor 488-conjugated goat anti-mouse secondary antibody (1:300) and Alexa Fluor 594-conjugated goat anti-rabbit secondary antibody (1:300) were added for 1 h at room temperature. Finally, the slides were stained with DAPI solution for 10 min and captured by a laser scanning confocal fluorescence microscope (Olympus FV 1000, Japan). Ten nonoverlapping high-power fields (40X) were randomly captured in each specimen and analyzed by ImageJ software.

### Western Blot Analysis

For western blot assays, renal tissues were lysed and homogenized in RIPA buffer supplemented with protease inhibitor cocktail l (C0001-1, Targetmol, China) and quantified with a BCA kit (P0013C, Beyotime, China). Protein sample extracts (30 mg/lane) were separated by SDS-PAGE and transferred onto a polyvinylidene difluoride membrane (PVDF). After the membranes were blocked with 5% BSA, they were incubated with the primary antibodies at 4°C overnight, followed by HRP-conjugated secondary antibody. Then, the membranes were incubated with HRP-conjugated secondary antibody (Boster Biological Technology Co., Ltd., China) at room temperature for 1 h. Films were scanned by a ChemiScope 6,000 system (Qinxiang, Shanghai, China). ImageJ software was used to measure the protein bands based on that of GAPDH.

### Immunofluorescence Staining

Frozen sections were used to assess colocalization of Pink1 and TOM20. Kidney sections were blocked with 5% bovine serum albumin for 30 min at room temperature, followed by incubation overnight at 4°C with primary antibodies against Pink1 (1:200, SC517353, Santa Cruz, United States) and TOM20 (1:200, 11802-1-AP Proteintech, United States). After the samples were washed with PBS, an Alexa Fluor 488-conjugated goat anti-mouse secondary antibody (1:300) and Alexa Fluor 594-conjugated goat anti-rabbit secondary antibody (1:300) were added for 1 h at room temperature. Finally, the slides were stained with DAPI solution for 10 min and captured by a laser scanning confocal fluorescence microscope (Olympus FV 1000, Japan). Ten nonoverlapping high-power fields (40X) were randomly captured in each specimen and analyzed by ImageJ software.

### Statistical Analysis

GraphPad Prism software was used for statistical analysis. Quantitative data are expressed as the mean ± standard error of the mean (SEM). One-way ANOVA was used for all experimental data followed by Dunnett’s test. A *p* value <0.05 was considered significant.

## Results

### Identification of the Chemical Components in Tongluo Yishen Decoction

**FIGURE 1:**
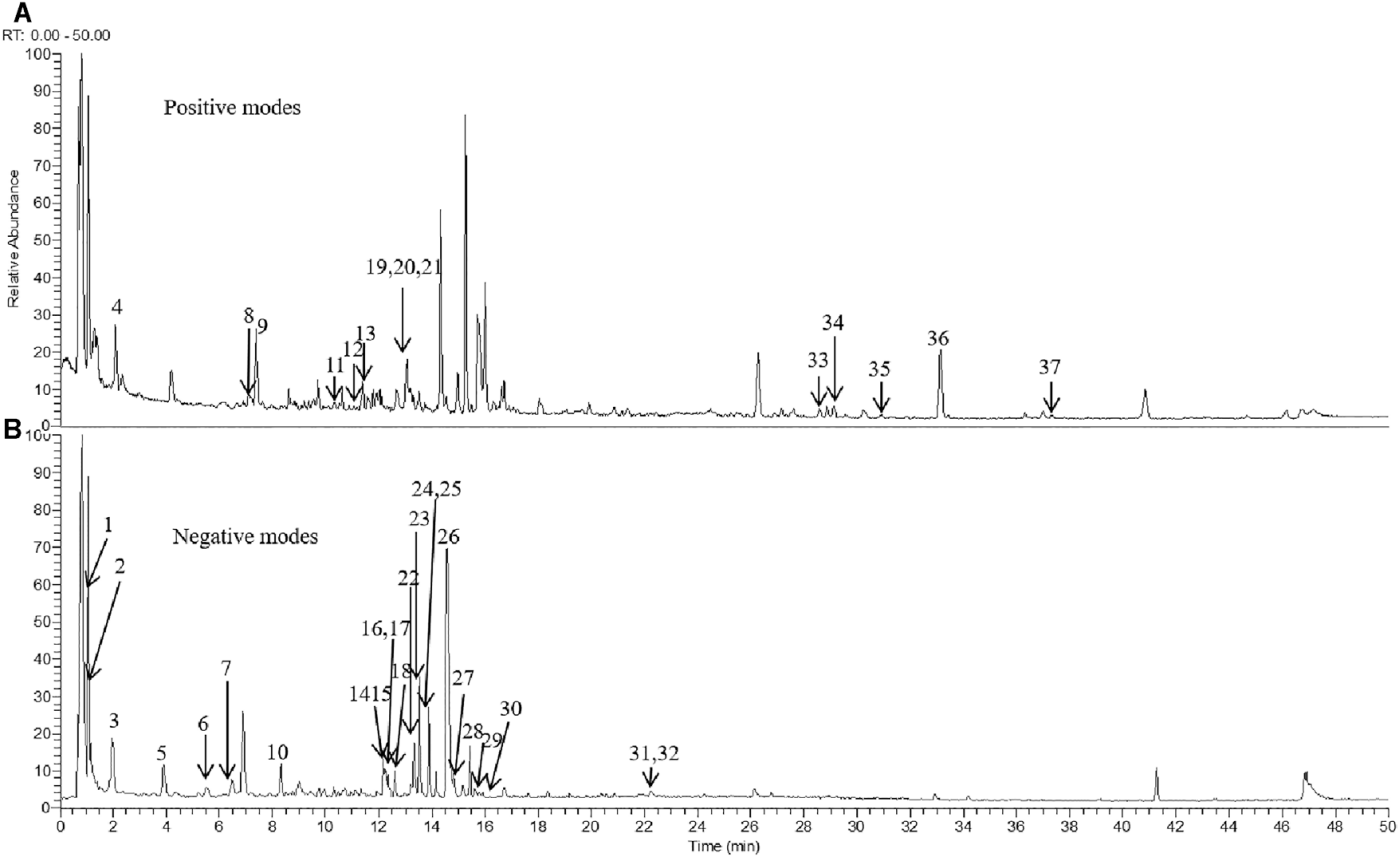
TLYS samples were examined by UHPLC–MS. Total ion chromatography in positive **(A)** and negative **(B)** ion modes for TLYS samples are shown.

To evaluate the major chemical components, we analyzed TLYS decoction using UHPLC-MS in positive and negative ion mode (Figure 1). Thirty-seven compounds (6 organic acids, 5 diterpene quinones, 4 sterones, 6 flavonoids, 10 phenolic acids and 6 other compounds) were detected at relatively high levels. The detailed information is shown in Table 2.

**TABLE 2:** Components identified in TLYS decoction.

Peak NO.	*t*R/min	Moleculear formulla	Dection pattern	m/z	Secondary debris (MS/MS)	Source	Identification	CAS
1	0.83	C_6_H_8_O_7_	−	191.01950	111, 87, 85	Unknown	Citric acid or isocitric acid	77-92-9 or 320-77-4
2	1.05	C_6_H_8_O_7_	−	191.01950	111, 87, 85	Unknown	Citric acid or isocitric acid	77-92-9 or 320-77-4
3	1.96	C_9_H_10_O_5_	−	197.04533	179, 135, 123	*Salvia miltiorrhiza* Bge	Danshensu	76822-21-4
4	2.08	C_9_H_11_NO_2_	+	166.08610	120	Unknown	L-Phenylalanine	63-91-2
5	3.90	C_7_H_6_O_3_	−	137.02415	93	Unknown	4-Hydroxybenzoic acid	99-96-7
6	5.53	C_15_H_14_O_6_	−	289.07170	245, 203, 125	*Spatholobus suberectus* Dunn	Catechin	7295-85-4
7	6.47	C_9_H_8_O_4_	−	179.03477	135	*Salvia miltiorrhiza* Bge	Caffeic acid	331-39-5
8	7.13	C_33_H_38_O_23_	+	803.18799	303, 479	*Carthamus tinctorius* L	2-(3,4-dihydroxyphenyl-3-{[2-O-(β-D-erythro-hexopyranosyl)-β-D-glycero-hexopyranosyl] oxy}-5-hydroxy-4-oxo-4H-chromen-7-yl-β-D-threo-hexopyranosi-duronic acid	—
9	7.38	C_27_H_32_O_16_	+	613.17627	355, 313, 211	*Carthamus tinctorius* L	Safflomin A	78281-02-4
10	8.31	C_15_H_14_O_6_	−	289.07156	245, 203, 109, 125	*Spatholobus suberectus* Dunn	L-Epicatechin	490-46-0
11	10.31	C_33_H_40_O_21_	+	773.21393	303	*Carthamus tinctorius* L	Cyanidin 3-O-β-(2″-ecaf-feoyl-glucopyranosyl)-(1→2)-O-β-galactopyranoside	—
12	11.19	C_27_H_30_O_16_	+	611.16095	287	*Carthamus tinctorius* L	Rutin	153-18-4
13	11.33	C_21_H_20_O_12_	+	465.10281	303	*Carthamus tinctorius* L	6-Hydroxykaempferol-3-O-glucoside	—
14	12.17	C_27_H_44_O_7_	−	525.30650 [M + COOH]^-^	159, 319, 301, 83	*Achyranthes bidentata* Bl	β-Ecdysone	5289-74-7
15	12.26	C_27_H_30_O_15_	−	593.15110	285, 255, 227	*Carthamus tinctorius* L	Kaempferol-3-O-rutinoside	17650-84-9
16	12.33	C_27_H_22_O_12_	−	537.1037	109, 185, 295	*Salvia miltiorrhiza* Bge	Lithospermic acid	28831-65-4
17	12.42	C_27_H_44_O_7_	−	525.30650 [M + COOH]^-^	159, 319, 479	*Achyranthes bidentata* Bl	β-Ecdysone isomer	—
18	12.60	C_27_H_44_O_7_	−	525.30650 [M + COOH]^-^	159, 319, 479	*Achyranthes bidentata* Bl	β-Ecdysone isomer	—
19	13.03	C_27_H_44_O_7_	+	481.31573	171	*Achyranthes bidentata* Bl	Inokosterone	15130-85-5
20	13.09	C_27_H_30_O_15_	+	617.14709	−	*Carthamus tinctorius* L	Safflor Yellow A	85532-77-0
21	13.14	C_27_H_30_O_15_	+	595.16571	287	*Carthamus tinctorius* L	Kaempferol 3-rutinoside	17650-84-9
22	13.33	C_36_H_30_O_16_	−	717.14703	339, 321	*Salvia miltiorrhiza* Bge	Salvianolic acid E	142998-46-7
23	13.54	C_18_H_16_O_8_	−	359.07700	161	*Salvia miltiorrhiza* Bge	Rosmarinic acid	20283-92-5
24	13.87	C_26_H_22_O_10_	−	493.11349	295,185	*Salvia miltiorrhiza* Bge	Salvianolic acid A	96574-01-5
25	13.87	C_26_H_20_O_10_	−	537.10360 [M + COOH]^-^	293, 197, 135, 105	*Salvia miltiorrhiza* Bge	Salvianolic acid C	15841-09-3
26	14.59	C_36_H_30_O_16_	−	717.14612	339, 321	*Salvia miltiorrhiza* Bge	Salvianolic acid B	121521-90-2
27	14.82	C_26_H_22_O_10_	−	493.11380	109, 185, 295	*Salvia miltiorrhiza* Bge	Salvianolic acid A isomer	—
28	15.43	C_36_H_30_O_16_	−	717.14673	321, 339, 519	*Salvia miltiorrhiza* Bge	Salvianolic acid Y	1638738-76-7
29	15.61	C_26_H_22_O_10_	−	493.11400	109, 185, 295	*Salvia miltiorrhiza* Bge	Salvianolic acid A isomer	—
30	15.75	C_19_H_18_O_8_	−	373.09310	135, 179	*Salvia miltiorrhiza* Bge	Methyl rosmarinate	99353-00-1
31	22.28	C_42_H_66_O_14_	−	793.43870	631, 569, 455, 113	*Achyranthes bidentata* Bl	Chikusetsu saponin IVa	51415-02-2
32	22.28	C_47_H_72_O_20_	−	955.45600	835, 793, 631, 455, 161	*Achyranthes bidentata* Bl	Achyranthoside G	—
33	28.59	C_19_H_20_O_3_	+	297.14822	253, 238, 211	*Salvia miltiorrhiza* Bge	Cryptotanshinone isomer	—
34	29.12	C_18_H_14_O_3_	+	279.10147	261,233	*Salvia miltiorrhiza* Bge	Dihydrotanshinone I	87205-99-0
35	31.27	C_19_H_18_O_3_	+	295.13306	90, 277, 249, 225	*Salvia miltiorrhiza* Bge	Tanshinone IIA isomer	—
36	33.14	C_19_H_20_O_3_	+	297.14822	251, 254, 279, 282	*Salvia miltiorrhiza* Bge	Cryptotanshinone	35825-57-1
37	37.32	C_19_H_18_O_3_	+	295.13306	277, 249, 235, 90	*Salvia miltiorrhiza* Bge	Tanshinone IIA	568-72-9

### The Effect of Tongluo Yishen Decoction on Renal Function and Histological Injury

**FIGURE 2:**
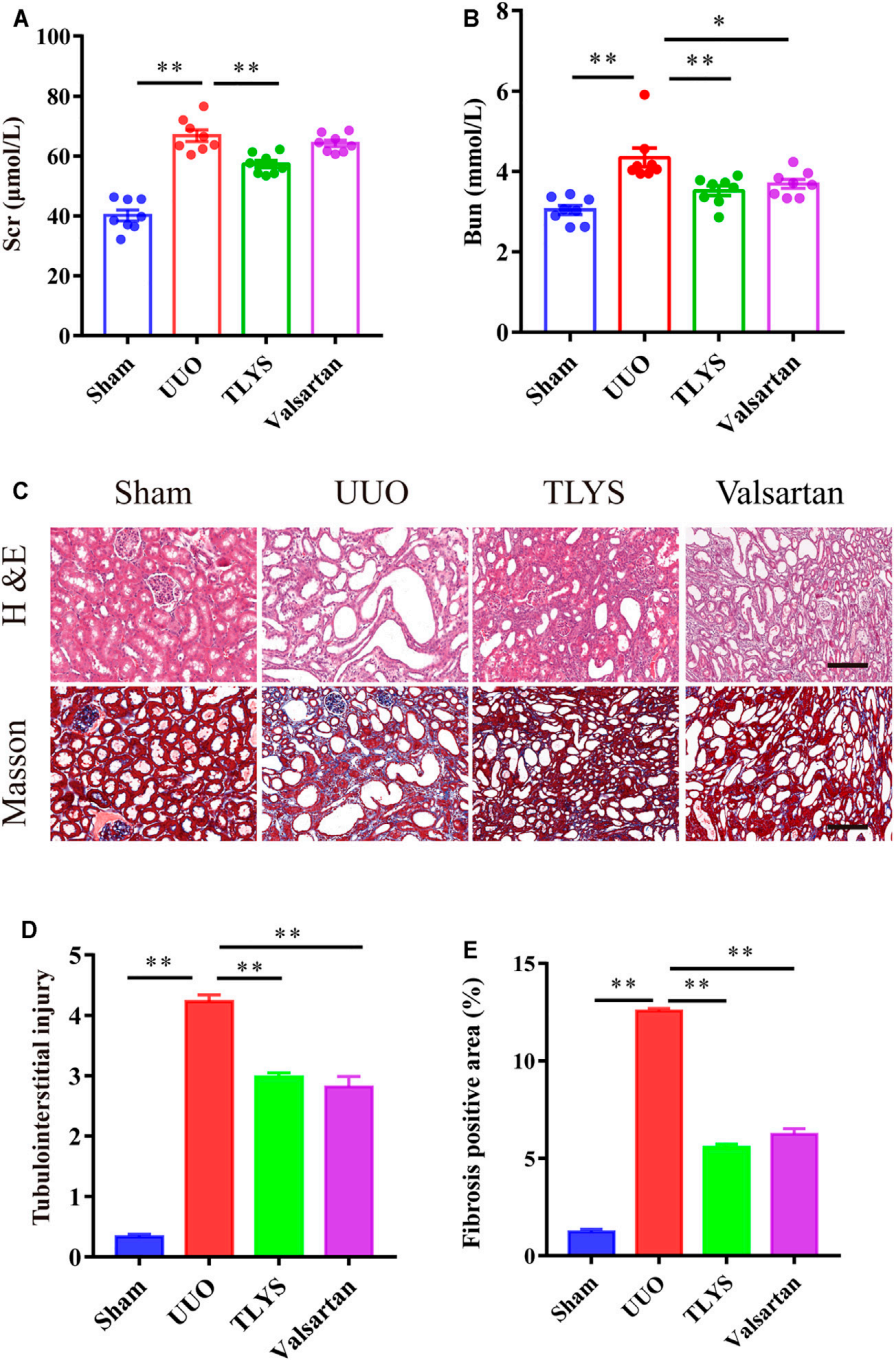
TLYS decoction alleviates renal function and pathological kidney injuries in UUO rats. **(A,B)** serum creatinine and BUN were detected (*n* = 8). **(C)** H&E and Masson’s trichrome were performed to evaluate kidney injury (*n* = 6). **(D)** Tubular damage scores based on H&E staining. **(E)** quantification of collagen areas according to Masson’s trichrome staining. The magnification of the images is ×200, scale bar = 50 μm. Data were presented as means ± SEM. *p < 0.05, **p < 0.01.

To study the effects of TLYS decoction on renal fibrosis, we adopted a rat model of UUO with 14-days TLYS treatment. We found that in the UUO group, the level of Scr was significantly higher than that in the sham group (66.79 ± 1.93 μmol/L vs 40.14 ± 1.83 μmol/L), and TLYS treatment partly decreased the levels to 57.25 ± 1.20 μmol/L (Figure 2A). Similarly, the rats with UUO showed significantly higher levels (4.36 ± 0.23 mmol/L) of BUN than the control rats (3.04 ± 0.11 mmol/L), while TLYS treatment significantly reduced the BUN levels to 3.52 ± 0.12 mmol/L (Figure 2B). As a positive control group, losartan (30 mg/kg) decreased serum concentration of BUN (*p* < 0.05) but not Scr. H&E staining and Masson’s trichrome staining were used to evaluate renal pathological injury (Figure 2C). H&E staining showed that the UUO group exhibited notable tubule atrophy and lumen dilation with diffuse interstitial inflammation. TLYS treatment attenuated kidney tubulointerstitial injury following UUO whereas the positive control, losartan, decreased the tubulointerstitial injury (Figure 2D). Masson’s staining showed substantial interstitial fibrosis in the UUO group; this fibrosis was significantly attenuated in the TLYS-treated rats (Figure 2E). These data suggested that treatment with TLYS could significantly mitigate renal injury.

### The Effect of Tongluo Yishen Decoction on Renal Fibrosis

**FIGURE 3:**
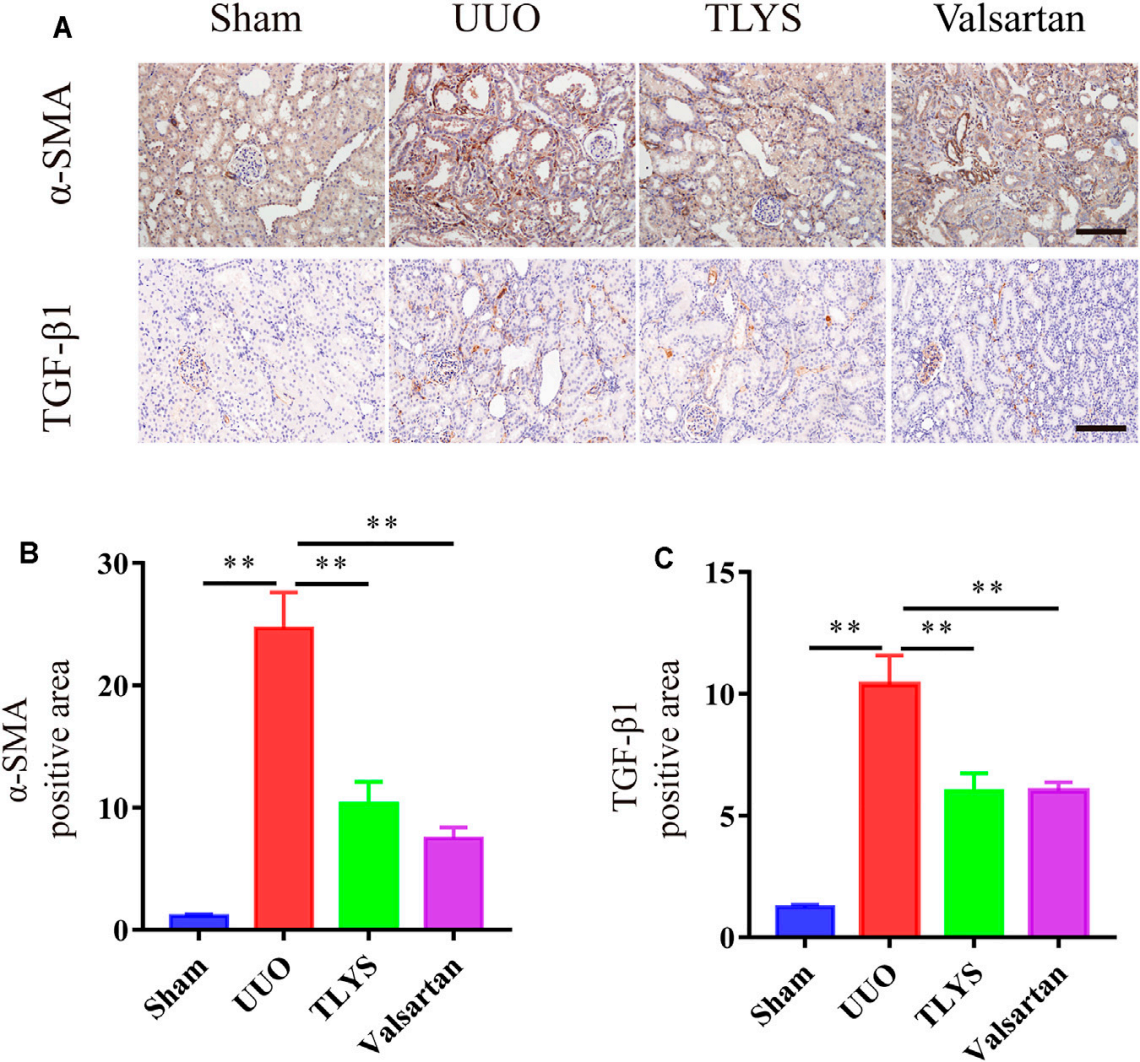
TLYS decoction suppresses a-SMA expression and TGF-β1 expression in UUO rats. **(A–D)** Expression levels of a-SMA and TGF-β1 in the kidney were detected by immunohistochemistry and analyzed the optical intensity of the abovementioned proteins was measured (*n* = 6). The magnification of the images is ×200, scale bar = 50 μm. Data were presented as means ± SEM (*n* = 6). *p < 0.05, **p < 0.01.

Given the protective effect of TLYS on renal fibrosis, we investigated the expression of α-SMA and TGF-β1 by immunohistochemistry. As shown in Figures 3A,B, the expression of α-SMA in the tubular interstitium was much higher in the UUO group than in the sham group. However, in the TLYS group, this increase was significantly suppressed. In addition, compared with the sham group, the UUO group showed a dramatic increase in TGF-β1 levels, while TLYS treatment significantly inhibited this abnormal increase in TGF-β1 (Figures 3A,C). Similarly, valsartan treatment significantly reduced the level of α-SMA and TGF-β1 (Figures 3A–C).

### Tongluo Yishen Decoction Showed Comprehensive Regulatory Effects in the Rats With UUO

**FIGURE 4:**
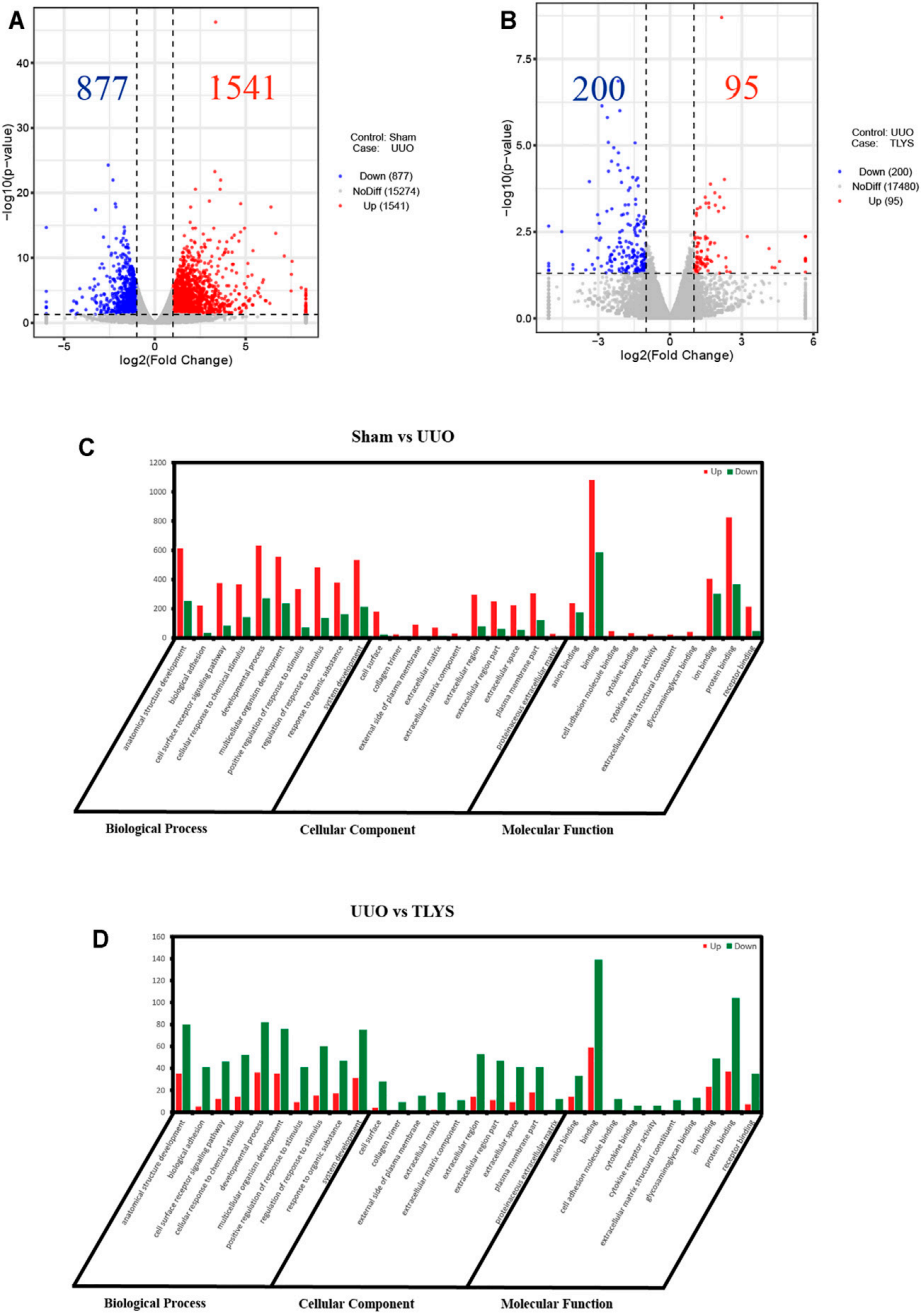
TLYS decoction played comprehensive regulatory roles in UUO rats. **(A,B)** Volcano plot of genes with significant differences, FDR <0.05, |log FC|> 1. **(C,D)** Gene Ontology (GO) function analysis of genes with significant differences in three experimental groups. Three independent samples were tested in each experimental group.

To explore the mechanisms of TLYS decoction, we performed RNA-Seq analysis. There were 1,541 DEGs with upregulated expression and 877 DEGs with downregulated expression in the UUO group relative to the sham group. Ninety-five DEGs had upregulated expression and 200 DEGs had downregulated expression in the TLYS group relative to the UUO group. Additionally, the differences were significant (|log2 (fold-change)| > 1 and *p* < 0.05, Figures 4A,B). Next, these overlapping DEGs were analyzed by GO analysis of biological processes, cellular components, and molecular functions. The number of DEGs with upregulated expression was significantly higher, whereas the number of DEGs with downregulated expression was lower in the UUO group than in the sham group (Figure 4C). After TLYS treatment, the number of DEGs with downregulated expression significantly increased and was greater than that of DEGs with upregulated expression (Figure 4D). These results further showed that TLYS has a comprehensive regulatory effect in the rats with UUO.

**FIGURE 5:**
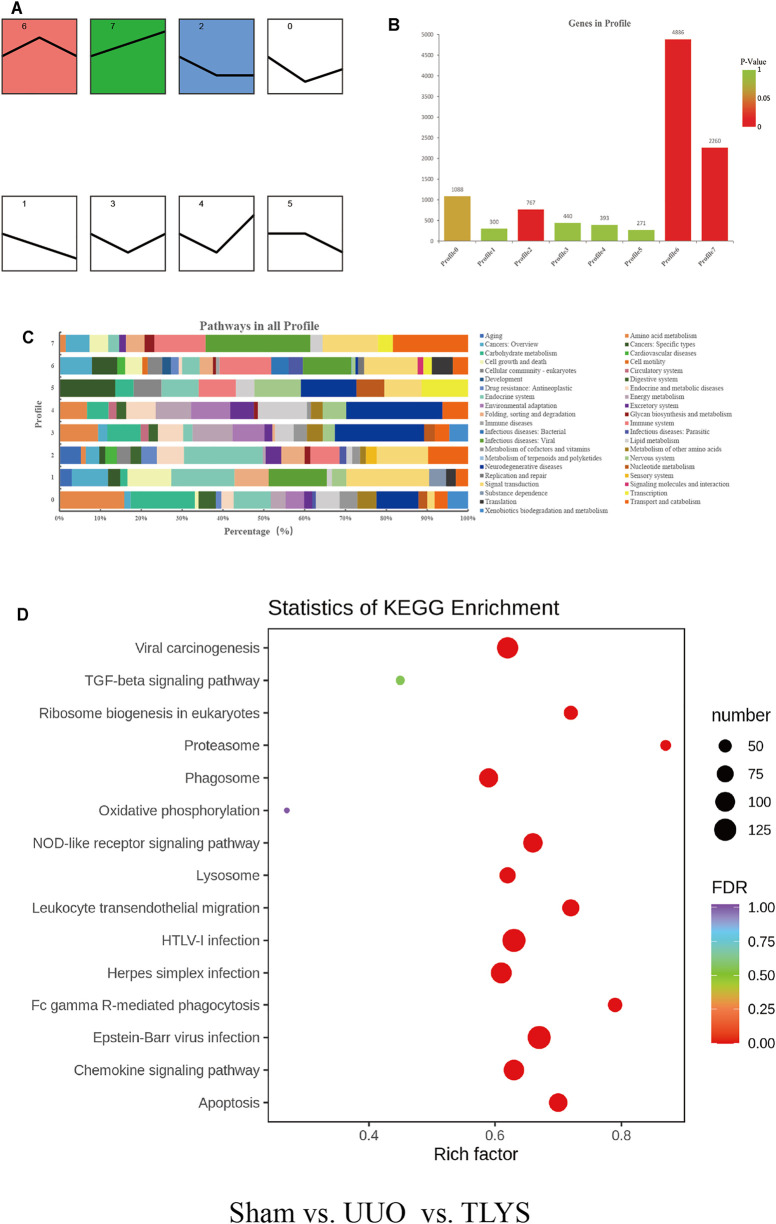
TLYS decoction may be related to mitochondria and mitophagy in UUO rats. **(A)** All trends (profile 0–7) of genes with significant differences. **(B)** Histogram of the four trends (red) with significant differences, *p* < 0.05. **(C)** Pathway-related gene distribution (%) in all profiles. **(D)** Bubble diagram of Kyoto Encyclopedia of Genes and Genomes (KEGG) pathway enrichment. Three independent samples were tested in each experimental group.

To screen out the most representative DEG group affected by TLYS, we performed a trend analysis of all selected DEGs. DEGs were divided into 8 categories (Figure 5A), of which profiles 2, 6 and 7 were significant (Figure 5B). We calculated the proportion of each DEG trend in the corresponding pathways, and signal transduction was commonly affected by the above three DEG trends (Figure 5C). Then, by KEGG enrichment analysis, three significant pathways were identified for TLYS treatment; the pathways were phagosomes (a membrane source in the earlier stages of autophagy), lysosome (an important component of autolysosome), and oxidative phosphorylation, which may participate in mitochondrial function (Figure 5D). Therefore, TLYS treatment might be associated with mitochondrial dysfunction and mitophagy in the rats with UUO.

### Tongluo Yishen Decoction Ameliorated Mitochondrial Function and Mitochondrial Dynamics

**FIGURE 6:**
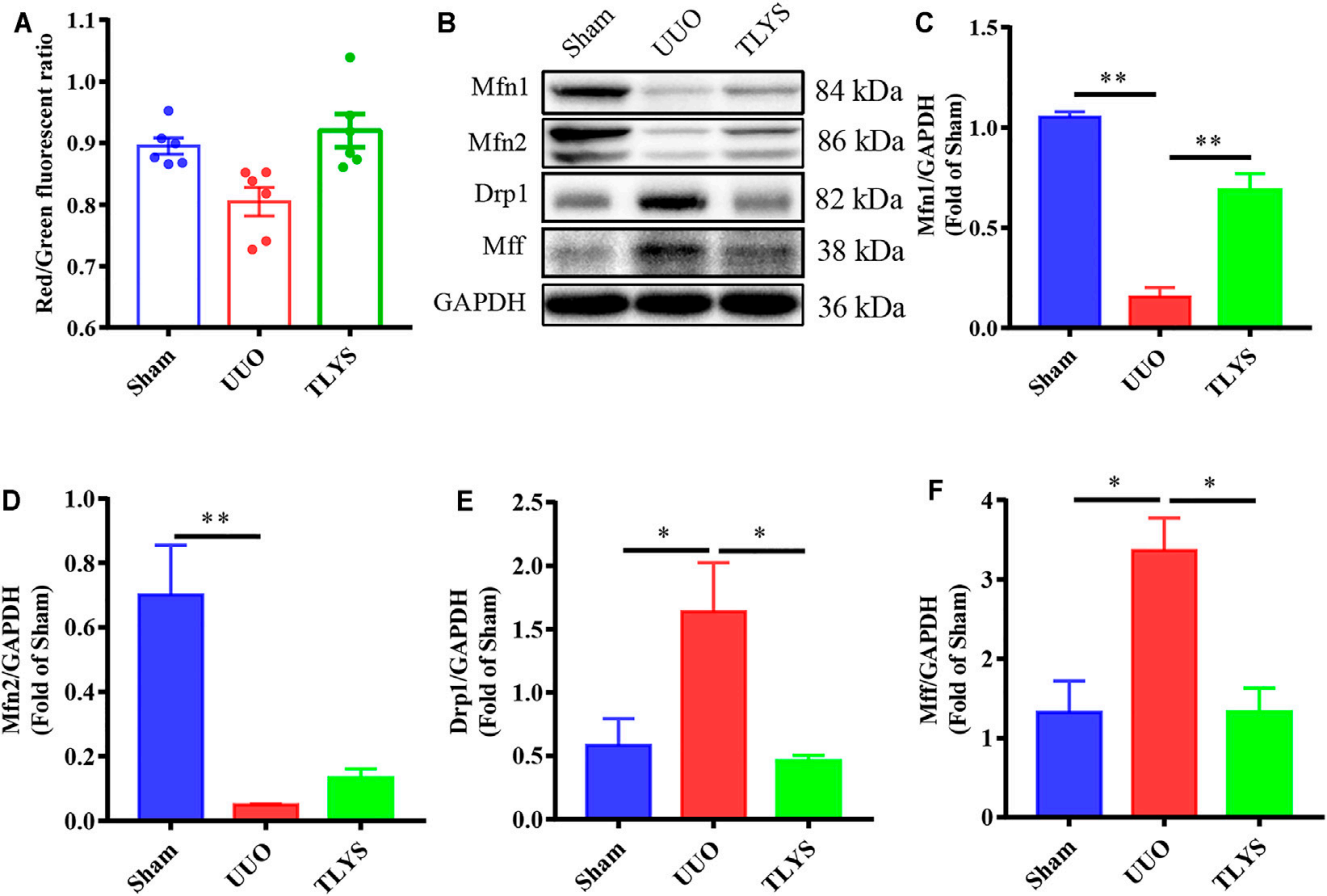
TLYS decoction attenuated mitochondrial function and dynamics in UUO rats. **(A)** Mitochondrial membrane potential (MMP) assessed using a JC-1 fluorescence probe (*n* = 6). **(B–F)** The protein levels of Mfn1, Mfn2, Drp1, and Mff were assayed by Western blot and analyzed semi-quantitatively. Data were presented as means ± SEM. *p < 0.05, **p < 0.01.

A reduction in MMP suggests damage to mitochondrial function, which is indicated by a lower ratio of red-to-green fluorescence. The MMP was lower in the UUO group than in the sham group, but TLYS increased the MMP level (Figure 6A). Mitochondrial division and fusion are crucial for maintaining morphology and function. The expression of proteins related to mitochondrial fusion (Mfn1 and Mfn2) showed a significant reduction in the UUO group (*p* < 0.01), and this trend was reversed after TLYS treatment (*p* < 0.01) (Figures 6B–D). As shown in Figures 6B,E,F, the expression of the proteins related to mitochondrial fission (Drp1 and Mff) was upregulated in the UUO group compared with the sham group, and TLYS treatment significantly prevented this trend.

### Tongluo Yishen Decoction Ameliorated Oxidative Stress in the Rats With UUO

**FIGURE 7:**
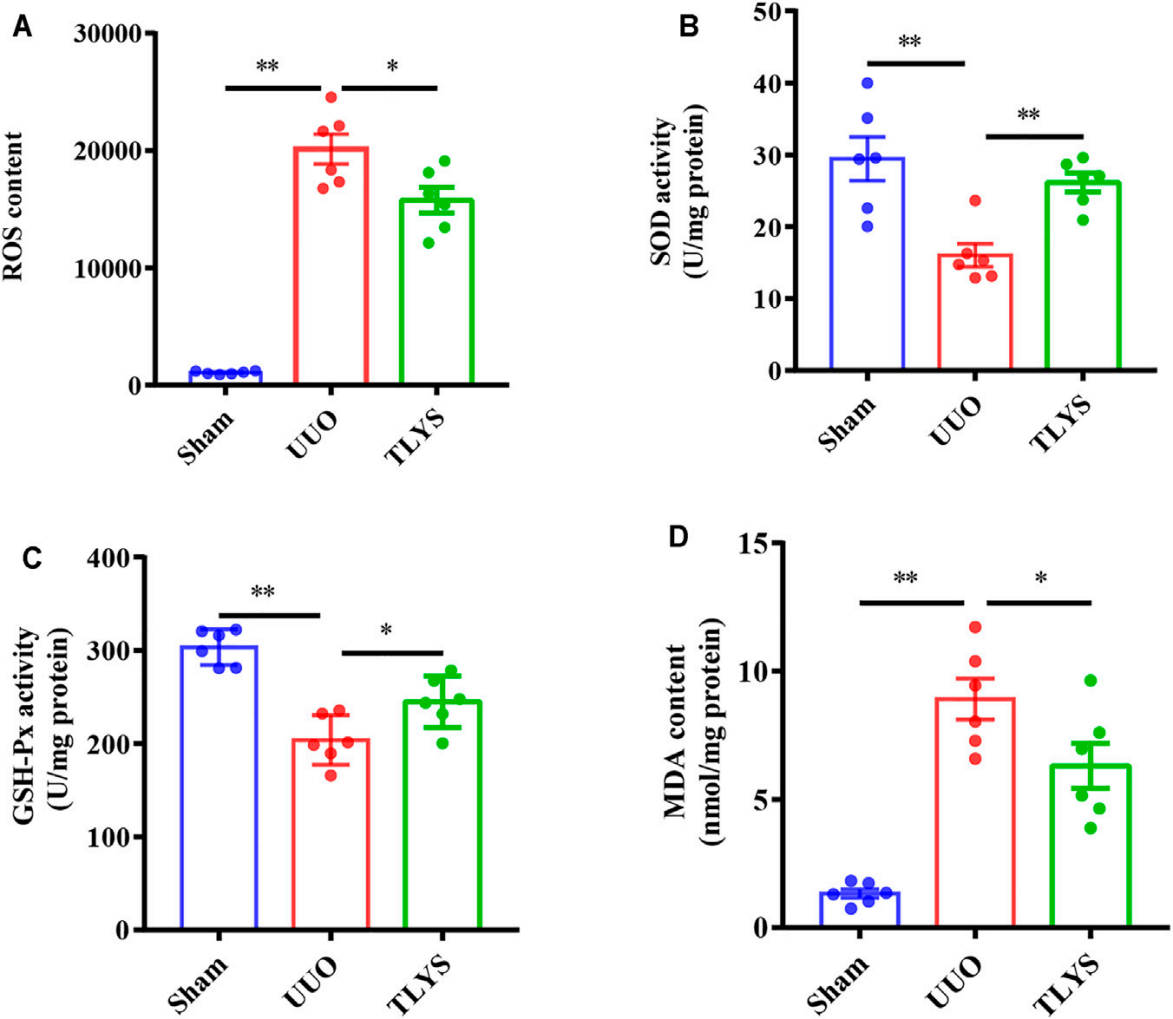
TLYS decoction ameliorated oxidative stress in UUO rats. The renal tissues were taken to evaluate the contents of ROS **(A)**, superoxide dismutase (SOD) **(B)**, glutathione peroxidase (GSH-Px) **(C)**, and malondialdehyde (MDA) (*n* = 6). Data were presented as means ± SEM. *p < 0.05, **p < 0.01.

Oxidative stress induced by the accumulation of ROS causes serious damage to mitochondria; therefore, we investigated the effects of TLYS on oxidative stress. Our results demonstrated that the activities of SOD and GSH-PX were significantly lower and that the content of ROS and MDA was higher in the UUO group than in the sham group. Interestingly, TLYS treatment significantly enhanced the levels of SOD and GSH-PX and inhibited the increase in ROS and MDA (Figures 7A–D). The results indicated that TLYS could ameliorate oxidative stress in the rats with UUO.

### Tongluo Yishen Decoction Alleviated Pink1/Parkin-Mediated Mitophagy in the Rats With UUO

**FIGURE 8:**
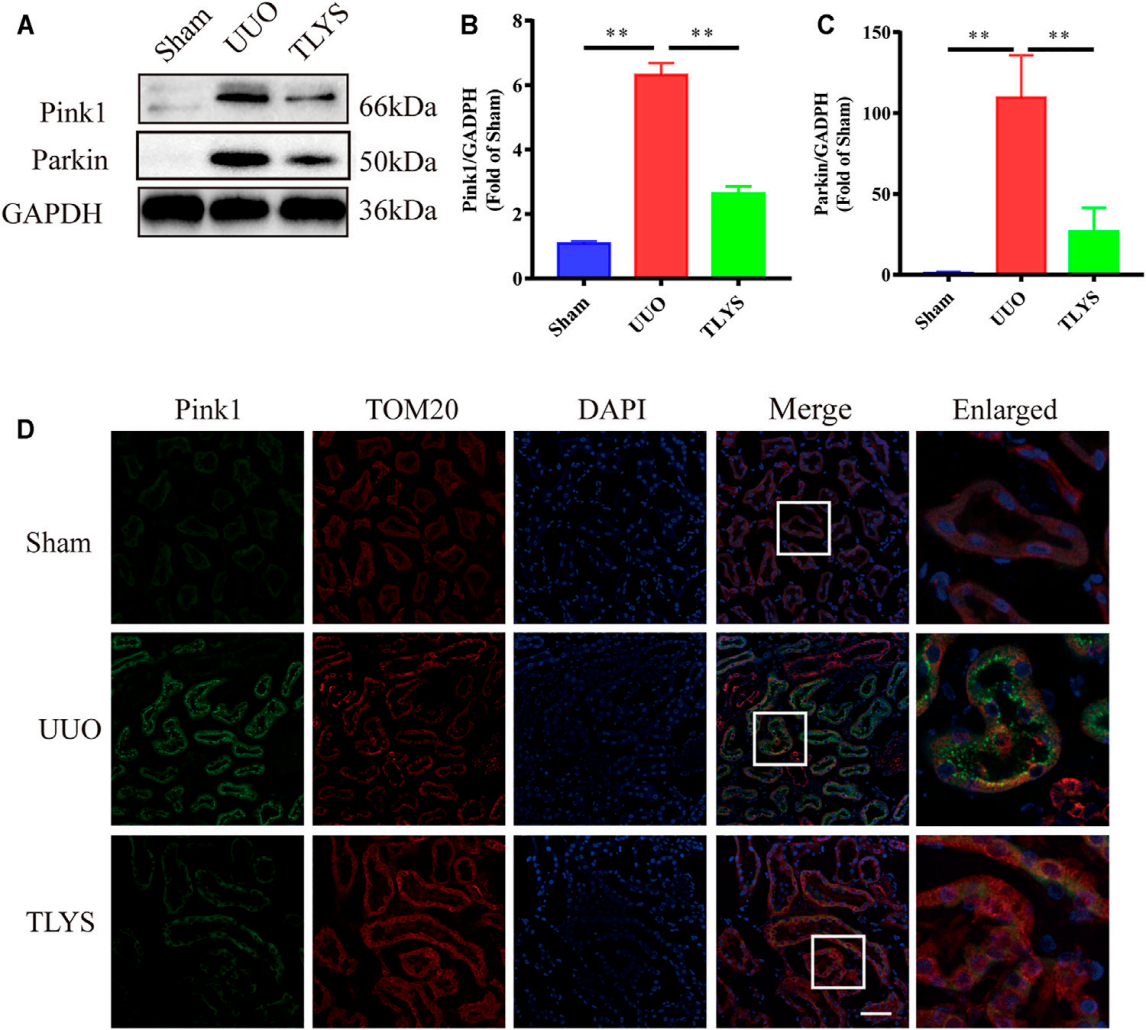
TLYS decoction alleviated Pink1/Parkin-mediated mitophagy in the rats with UUO. **(A-C)** The expression of Drp1, Pink1, Parkin and Mfn2 by Western blot (*n* = 6) and analyzed semi-quantitatively. **(D)** Images of tissues co-stained for the mitochondrial markers Pink1 (green), TOMM20 (red) and nuclear (blue), scale bar = 50 μm, (*n* = 6). Data were presented as means ± SEM. *p < 0.05, **p < 0.01.

Damaged mitochondria can be degraded by mitophagy, which is driven by Pink1/Parkin signaling. In our study, we assessed the protein expression of this pathway. Compared with the sham group, the UUO group showed significantly increased protein levels of Pink1 and Parkin in the kidney. However, TLYS treatment significantly decreased the expression of Pink1 and Parkin. Moreover, immunofluorescence confirmed and further revealed that Pink1 in the renal tubular epithelial cell was diminished in the rats with UUO compared with the sham rats (Figures 8A–C). The colocalization of mitochondria (marked by TOM20) and Pink1 was markedly decreased in the TLYS group compared with the UUO group (Figure 8D). These results suggested that the translocation of Pink1 from the cytoplasm to mitochondria machinery is inhibited, resulting in the accumulation of Pink1 at the outer mitochondrial membrane in the UUO group, and TLYS could reverse this change. These data indicated that TLYS inhibited Pink1/Parkin-mediated mitophagy in the rats with UUO.

## Discussion

Although the definite pathological mechanism of CKD remains unclear, RIF is regarded as the final common pathway of CKD leading to end-stage renal failure, without regard to etiology (Boor and Floege 2011). RIF is closely associated with a deterioration in renal function in patients with CKD (Nangaku 2006). Because the pathogenesis of RIF has not yet been fully clarified, effective and specific therapeutic methods for the treatment of RIF are lacking, and the development of strategies to prevent and intervene in RIF would be beneficial for patients with CKD. TLYS decoction is composed of four herbs, and 38 different compounds were identified by UHPLC-MS analysis. Studies have also shown that quercetin and salvianolic acid B can effectively improve RIF. Furthermore, in a model of acute pancreatitis, tanshinone IIA decreased ROS release and protected the mitochondrial structure (Chen et al., 2020). In addition, achyranthes was reported to have a protective effect on the kidney by reducing the accumulation of ROS and apoptosis in the renal tissues of mice with acute kidney injury (Wang et al., 2020). These protective effects and mechanisms are consistent with our observations in this study. These ingredients may be involved in the protective effect of TLYS in RIF. In this study, we investigated the renal protective effects of TLYS in rats with UUO and its underlying molecular mechanisms. Renal function is assessed by Scr and BUN, which reflect glomerular filtration barrier impairment and renal filtration function. In this study, we found that TLYS reduced the levels of Scr and BUN in the rats with UUO. This finding suggested that TLYS plays a crucial role in improving renal function in these rats. RIF is a dynamic and converging process characterized by activated tubulointerstitial myofibroblasts and ECM, and activated myofibroblasts are thought to be a major contributor to the pathogenesis of RIF (Nangaku 2006). α-SMA is a marker protein of myofibroblasts, and TGF-β1 is a key mediator in progressive renal fibrosis. TGF-β1 can enhance fibroblast growth and collagen production and promote the differentiation of fibroblasts into myofibroblasts, which secrete ECM components (Meng, Nikolic-Paterson, and Lan 2016). In this study, α-SMA and TGF-β1 levels were significantly lower in the TLYS group than in the UUO group, indicating that TLYS might alleviate RIF through its antifibrotic effect.

Next-generation high-throughput RNA-Seq is an unbiased technology that can objectively reveal gene expression changes in disease and reveal unknown transcripts that are not annotated in current databases (Trapnell et al., 2010). In this study, we found that three significant pathways (phagosomes, lysosome, and oxidative phosphorylation pathways) were affected in UUO by TLYS treatment. Then, we observed the abnormal mitochondrial function during UUO and found that it was improved by TLYS treatment, which might rescue mitochondrial function by inhibiting oxidative stress.

The increase in ROS in the cytoplasm triggers the opening of mitochondrial permeability transition pores and leads to dissipation of the MMP, inhibition of ATP production, and induction of mitochondrial swelling (Yang et al., 2017). Mitochondrial outer membrane fusion is mediated by Mfn1 and Mfn2, and the recruitment of Drp1 from the cytosol to the outer mitochondrial membrane is mediated by its receptor proteins (Mff), which are involved in mitochondrial division. Although in several CKD models, there is a shift of mitochondrial dynamics toward fission (Hallan and Sharma 2016; Xiao et al., 2017; Aparicio-Trejo et al., 2018), the involvement of this change in UUO is still under discussion. Our results indicated that there was mitochondrial damage (decreased MMP) and a dynamic imbalance of mitochondria (upregulated Drp1 and Mff expression, downregulated Mfn1 and Mfn2 expression) in the rats with UUO. Interestingly, TLYS treatment could preserve the stability of mitochondrial structures.

For mitochondrial damage, excessive oxidative stress causes the most serious damage to mitochondrial membrane permeability, especially lipid peroxidation of the inner membrane. Oxidative stress includes increasing levels of ROS and the loss of antioxidant enzymes, such as SOD and GSH-PX, which play crucial roles in protecting kidneys against oxidative stress. Previous studies demonstrated that MDA was significantly increased in rats with ureteral obstruction compared with sham-treated rats (Shi et al., 2020). ROS production and RIF are enhanced by deficiencies in these antioxidant enzymes (Aranda-Rivera et al., 2021). In our study, UUO led to a decrease in the activities of SOD and GSH-PX compared to those of the sham group. In contrast, the MDA level was markedly increased compared with that in the sham group. TLYS treatment mitigated the oxidative stress induced by UUO. Briefly, the above results indicated that TLYS may reduce mitochondrial damage through antioxidative stress in the rats with UUO. However, the abnormal mitochondria clearance and the regulatory effects of TLYS on mitophagy need further study.

Mitophagy, mediated by the Pink1/Parkin pathway, is a major mechanism to remove damaged mitochondria (Zhu et al., 2013). Autophagic flow is a dynamic process, and an increase in Pink1/Parkin levels does not indicate normal mitophagic flow, as illustrated by the increase in the number of damaged mitochondria and autophagic bodies in these models (Aparicio-Trejo et al., 2019; Avila-Rojas et al., 2019; Aparicio-Trejo et al., 2020), which could indicate disruption downstream of mitophagic flux. Therefore, it is not clear whether mitophagic flow is carried out properly in tubular epithelial cells in the rats with UUO due to the accumulation of damaged mitochondrial bodies observed by electron microscopy. In this context, Sang et al. (Sang et al., 2020)showed that the upregulation of renal calcineurin 1 induced translocation of Drp1 to the mitochondria, increasd mitochondrial fission, and regulated mitochondrial dynamics. However, the increase in mitochondrial fission, PINK1 and Parkin may also indicate mitophagic dysfunction. Similarly, our results indicated that there is an activation in Pink1/Parkin-mediated mitophagy, including increased Pink1 and Parkin levels. TLYS could decrease Pink1 and Parkin levels and alleviate the translocation of Pink1 from the cytoplasm to mitochondria. Overall, the above results show that overactivation in Pink1/Parkin-mediated mitophagy occurs in UUO rats, and TLYS can improve damaged mitochondria function via Pink1/Parkin-mediated mitophagy to protect against RTC (renal tubular cell) injury. However, autophagic activation has different effects in kidneys with different statuses or under different stress factors. Therefore, more studies of each of the steps of mitophagic flow are still needed to elucidate its role in kidney obstructive damage.

In conclusion, our study showed that TLYS decoction can ameliorate renal pathological damage and improve renal function in UUO rats. This renoprotective effect may be related to a reduction in oxidative stress; thus, TLYS can improve mitochondrial function and dynamics to protect against RTC injury.

## Data Availability

The data presented in the study are deposited in the NCBI repository, accession number PRJNA767261.
