# Supplementary figures and images for: Tongluo Yishen Decoction Ameliorates Renal Fibrosis via Regulating Mitochondrial Dysfunction Induced by Oxidative Stress in Unilateral Ureteral Obstruction Rats
Source: Front Pharmacol. 2021 Oct 12;12:762756. doi: 10.3389/fphar.2021.762756 (PMC8545824; doi:10.3389/fphar.2021.762756)

## Slide 1
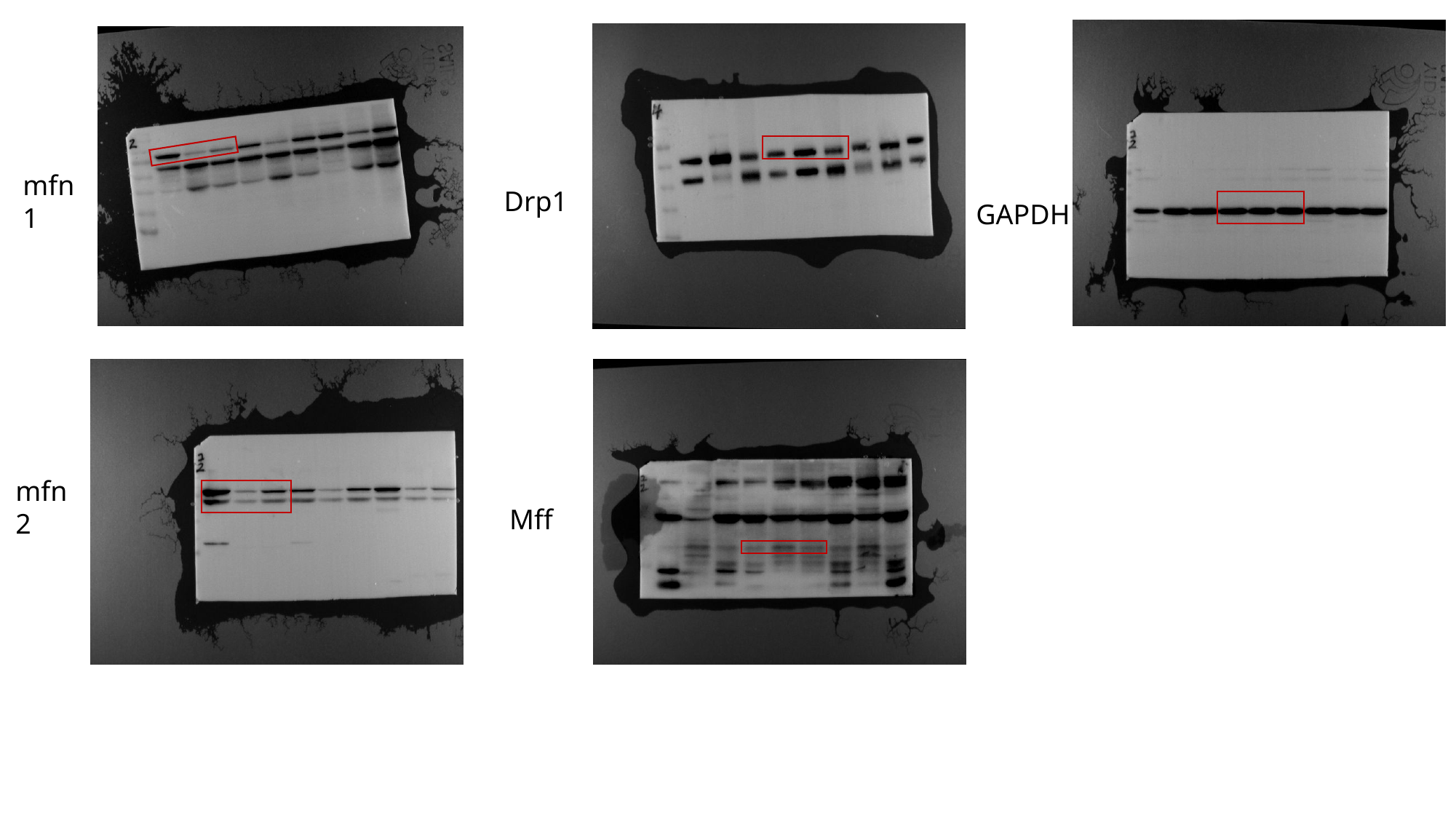

mfn1
Drp1
GAPDH
mfn2
Mff

## Slide 2
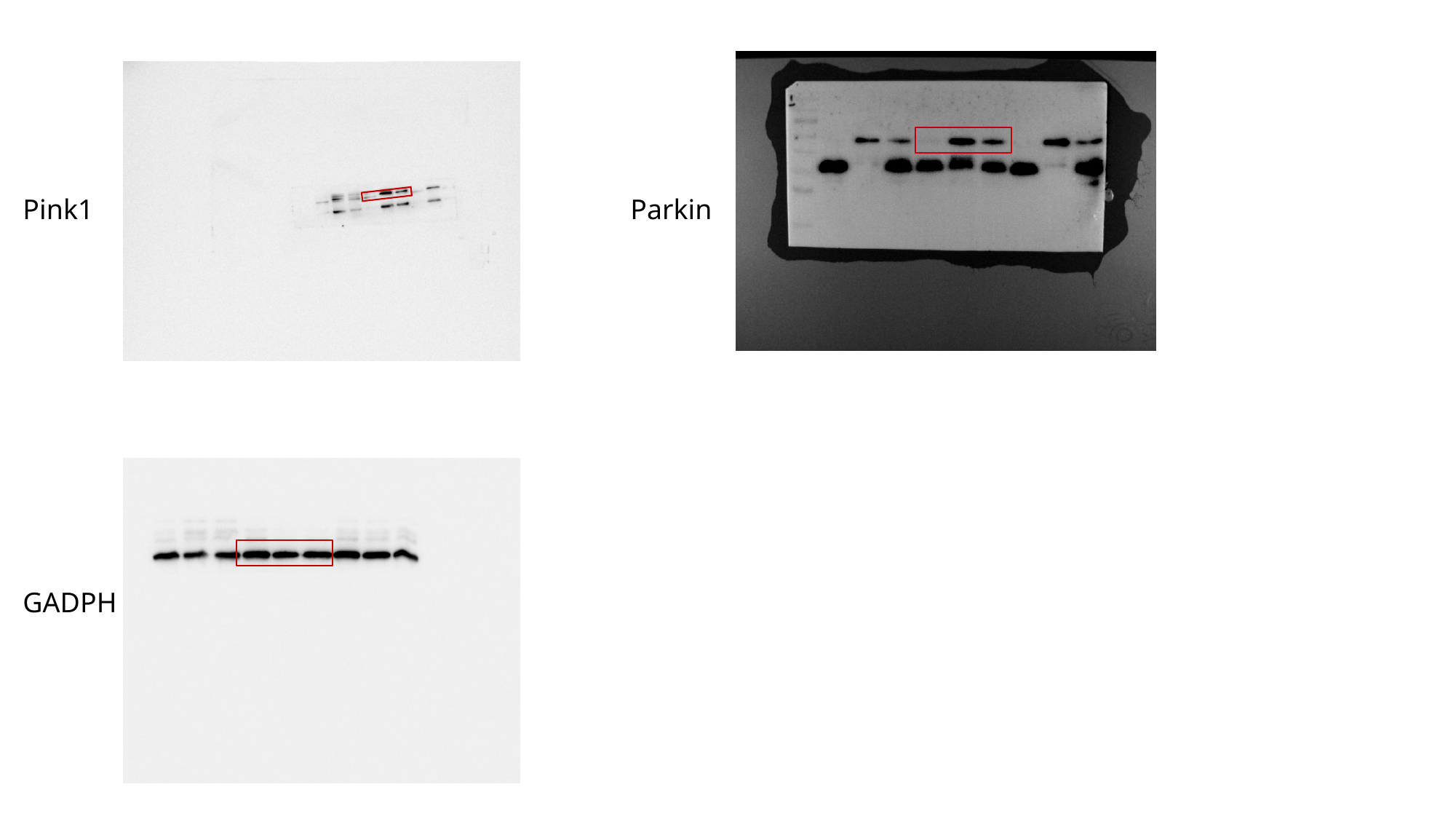

Pink1
Parkin
GADPH

Supplement: Supplementary file 1 [file Presentation1.PPTX]
